# Supplementary material for: Internal in-frame translation generates Cas11b, which is important for effective interference in an archaeal CRISPR-Cas system
Source: Front Microbiol. 2025 Feb 26;16:1543464. doi: 10.3389/fmicb.2025.1543464 (PMC11899642; doi:10.3389/fmicb.2025.1543464)
Supplement: Supplementary file 1 [file Data_Sheet_1.pdf]

Supplementary Material

Internal in-frame translation generates Cas11b, which is important for effective interference in an archaeal CRISPR-Cas system

Sailer, A.-L. †[1], Brendel, J. †[1], Chernev, A. [2,3], König, S. [2,3], Bischler, T. [4], Gräfenhan, T. [4], Urlaub, H. [2,3], Gophna, U. [5], Marchfelder, A. [1]*

Supplementary Figures

Supplementary Figure 1. Genes for CRISPR-Cas subunits in *H. volcanii* 2

Supplementary Figure 2. Confirmation of strain *cas8*bM545A by Southern blot analysis 3

Supplementary Figure 3. Interference test 4

Supplementary Figure 4. Peptides identified for Cas11b 5

Supplementary Figure 5. crRNA concentrations are not affected by Cas11b depletion 6

Supplementary Figure 6. Western blot analysis of gel filtration fractions 7

Supplementary Tables

Supplementary Table 1. Strains, plasmids and primers used 8

Supplementary Table 2. Regulation of pHV4 genes in a Cas11 less strain. 9

Separate Excel Tables

Supplementary Excel Table 3 includes the dataset for RNA-seq.

Supplementary Excel Table 4 includes mass spectrometry data.

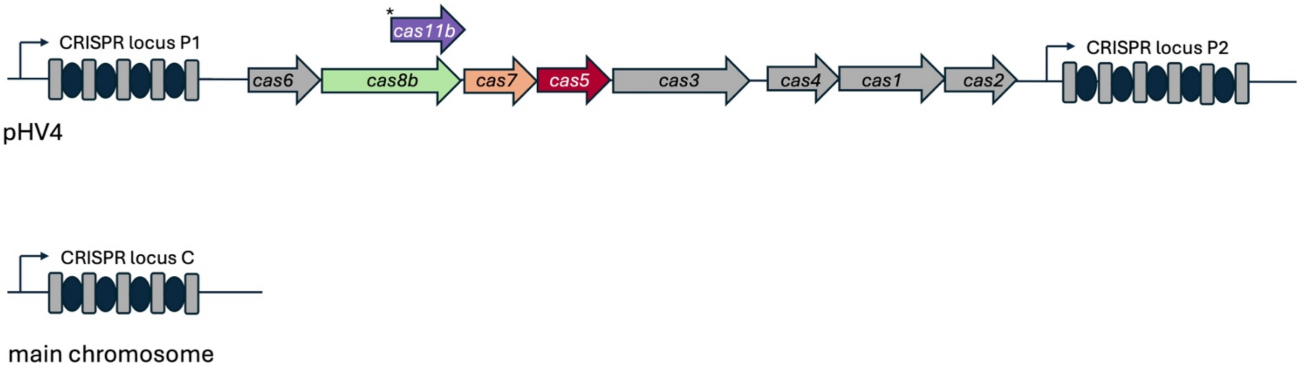

Supplementary Figure 1. Genes for CRISPR-Cas subunits in *H. volcanii*. The genes for the Cas proteins Cas1-8b and Cas11b are flanked by the two CRISPR loci P1 and P2, they are encoded on the megaplasmid pHV4. The third CRISPR locus C is encoded on the main chromosome. Cas11b is encoded in the 3´ part of the *cas8b* gene, the internal translation initiation site is marked with an asterisk.

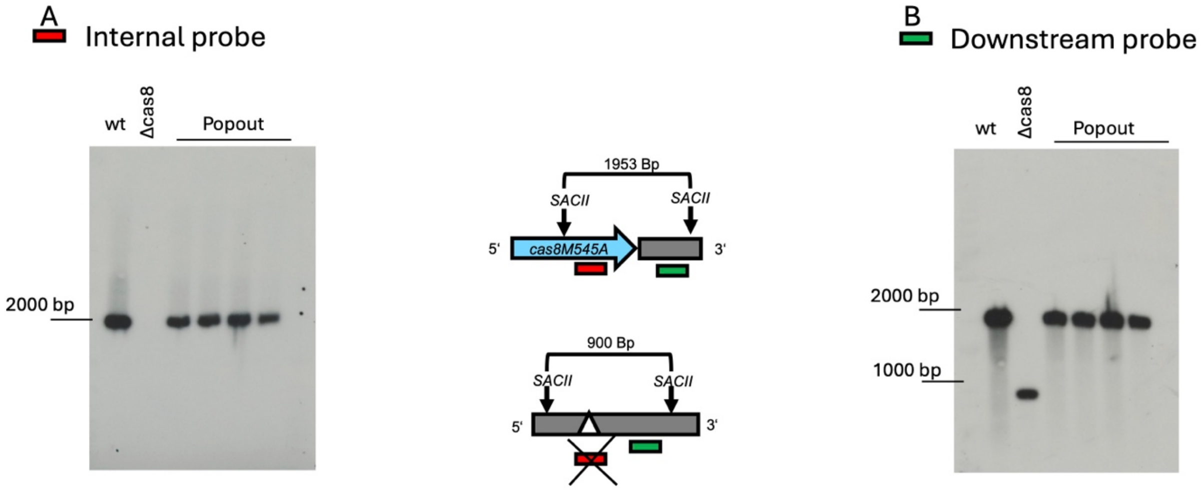

Supplementary Figure 2. Conformation of strain *cas8*bM545A by Southern blot analysis. To generate the mutant strain cas8M545A the mutated *cas8b* gene was re-integrated into the original genomic position of *cas8b* in a Δ*cas8b* strain. Four potential *cas8*M545A (HV120) clones were selected and checked for the presence of the mutated *cas8b* by Southern blot analysis. To this end, *Sac*II digested gDNAs were separated on an 0.8 % agarose gel and transferred to a nylon membrane by capillary blot. The membrane was hybridised with radioactively labelled PCR probes. **A.** A probe against the *cas8*M545A gene was used. **B.** A probe binding to the downstream region of the gene was used. A schematic representation of the expected sizes of the fragments bound by the probes (red for the internal probe and green for the downstream probe) is shown in the middle. The corresponding autoradiographs are shown at the sides, sizes of the 1 kb ladder are indicated on the left of the films.

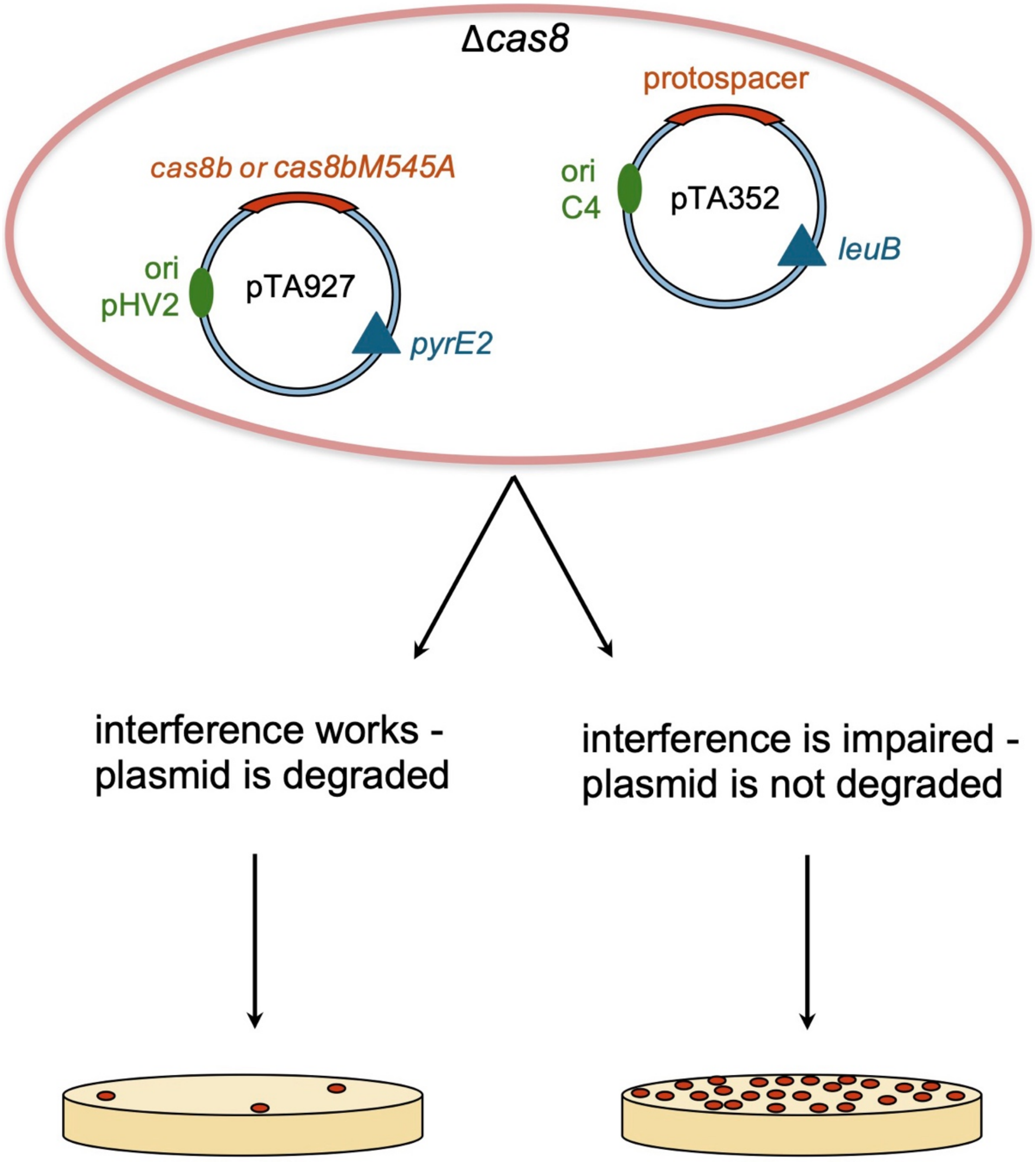

Supplementary Figure 3. Interference test. To test the activity of the CRISPR-Cas system an interference assay was used. The *Haloferax* strain Δ*cas8b* was transformed with a plasmid expressing either Cas8b and Cas11b (*cas8b*) or only Cas8b (mutant *cas8bM545A*). Cells were subsequently transformed with the "invader" plasmid, that contains a DNA fragment (protospacer) to which an endogenous crRNA can bind. This crRNA guides the Cascade complex to the plasmid and triggers degradation of the plasmid by Cas3. If the CRISPR-Cas system is active and interference works, cells cannot grow on selective media since the plasmid has been degraded. If Δ*cas8* is transformed with a *cas8b* gene, that expresses Cas8b and Cas11b, full interference activity is observed. However, if Δ*cas8b* cells are transformed with the mutant gene *cas8bM545A*, that only express Cas8b but not Cas11b, interference activity is clearly reduced.

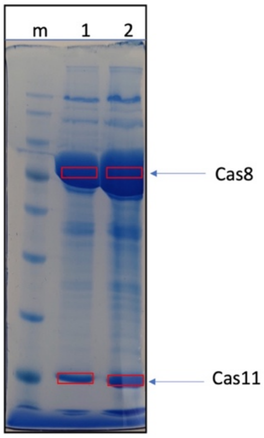

A.

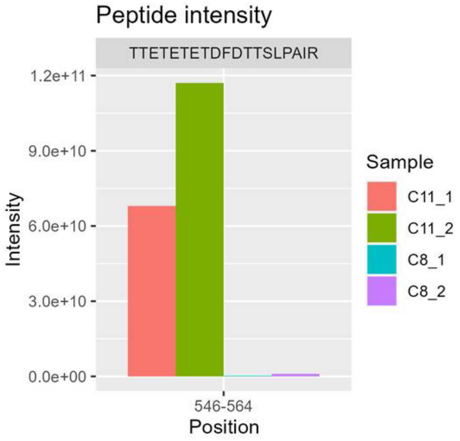

B.

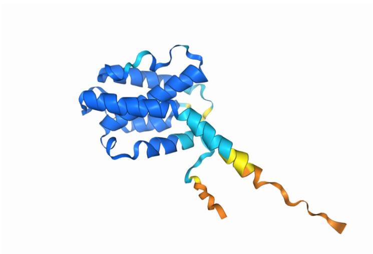

C.

D.

```
MTGPDIDDFENALNAFWHGRPPASLEDVMALYGVLAVAESGGELYGTDSK
LEPFVDDGRLVTIDIDLTGETPNVSDPKVDTLRVEDVSKLRYAHKSSGRG
AKYSLTQIGSKNGNDAEGVASTILGRVRSWTTQDSVRSVTGEDGHPDGWI
VEELAAVFEKGSDTLEALEEAIKALLPPDESLPTVITVRLRLDAGRLSHG
EESGPRWFWPAELDVLEEAMKRYATANAADKNVESGDGISEGESVGLVTD
RVERVVGTPDNPIGVFSVKHPDAQPGLRQDQSWRNYPVGADTAMLFSKGQ
DLVETCVLRRGGVETYALPYFAGELTPLKAQSLYGAIQSLDRESDYDDSG
GSPLARVTYELRESDDETLQELAKTELRFYTITLPIGDDKNVIAEEPAAP
VYWVSELADALAQTVHGPTLNPERGGFAPYDNWSLLELATEDFEESRKFG
FYRIVGHQFTDSAFAYRGDDEDDDFRRVVDHRLIAGVPLDASMLFDEYLR
RYHDESEGGDLPPHQIVAQQLVHLETLSRAGLLNGLDVPIEPPTMTTETE
TETDFDTTSLPAIREHRLESFLDRPLFEAPARRAAALAGVLVGQVSWHQE
SERNVGRPLDAQTKGDQLTKNSLENALTSALEKAKVYALDSEYRSDRDML
FPETVDRLLETTEDMPSAWPIEKRELQFCYVLGHAHGRRSMPVAFDLHEK
EDEDDQDTEEPAESTTN
```

Supplementary Figure 4. Peptides identified for Cas11b and structures (primary and tertiary) of Cas11b. A. Proteins identified as Cas8b and Cas11b in the Western blot (Figure 1) were isolated from Coomassie stained SDS gels from two different lanes (red boxes) yielding samples C11_1, C11_2 for Cas11b, and C8_1 and C8_2 for Cas8b. **B.** If translation initiation starts at M545 the first peptide of Cas11b is MTTETETETDFDTTSLPAIR. Peptide TTETETETDFDTTSLPAIR was found with clearly higher intensity in the Cas11b samples, suggesting that Cas11b translation is initiated at M545. The N-terminal M is very often removed after translation in haloarchaea. The complete data set is listed in Supplementary Table 4. **C.** Structure of Cas11b as predicted by AlphaFold (https://alphafoldserver.com) (Abramson *et al*, 2024). **D.** Sequence of the Cas8b protein, the Cas11b protein present in the C-terminal part is underlined.

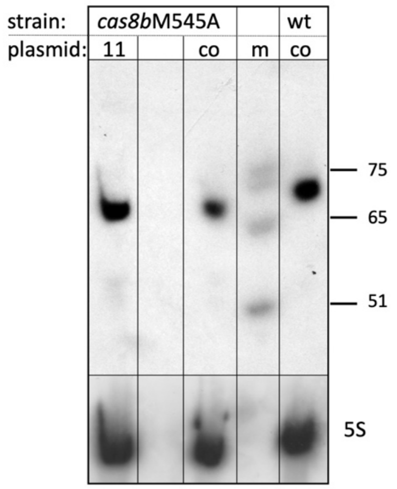

Supplementary Figure 5. crRNA concentrations are not affected by Cas11b depletion. In wild type *Haloferax* cells containing the *cas8b* gene on the chromosome (expressing Cas8b and Cas11b) crRNAs are readily visible (lane wt/ co; RNA from cells H119 x pTA927). The strain with the mutated *cas8b* gene (that does not express Cas11b) has similar amounts of crRNAs (lane *cas8*M545A/ co; RNAs from cells *cas8*M545A x pTA927). If the mutant strain is transformed with a plasmid expressing Cas11b again similar amounts of crRNAs are visible (lane *cas8*M545A/ 11; RNAs from cells *cas8*M545A x pTA927-*cas11b*). A DNA size standard is shown at the right, the upper panel shows hybridisation with a probe against a spacer of the P1 CRISPR locus, the lower panel shows hybridisation with a probe against the 5S rRNA.

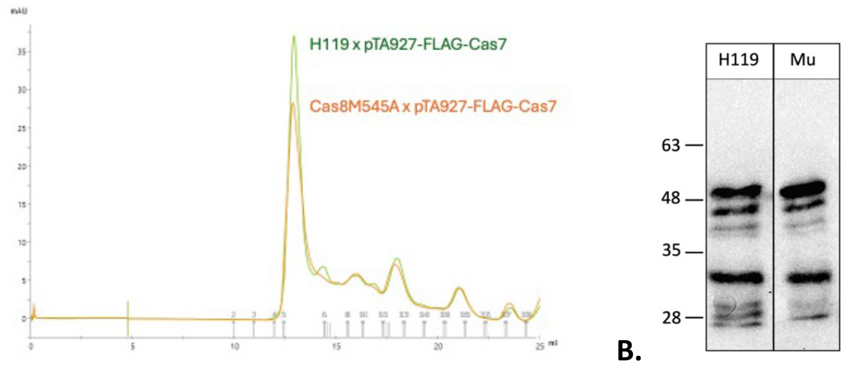

Supplementary Figure 6. Western blot analysis of gel filtration fractions. Cascade complexes from *Hfx. volcanii* can be purified via a FLAG-Cas7 co-purification with gel filtration as reported previously (Brendel, 2014). Cascade complexes from wild type cells (H119) and mutant cells *cas8*M545A, both transformed with a plasmid expressing FLAG-Cas7 were purified via FLAG-Cas7 using FLAG-agarose. Purified fractions were loaded onto a gel filtration column and fractions were collected. Proteins of fraction 6 from wild type cell and mutant cell extracts were loaded onto an SDS PAGE and subsequently transferred onto a Western membrane, which was hybridised with an antibody against the FLAG tag. The full length Cas7-FLAG fusion protein is visible at about 48 kDa. Due to the high amount of acidic amino acids, halophilic proteins run generally slower on SDS PAGE, adjusted to this the calculated molecular weight is 47.4 kDa (Brendel, 2014; Guan *et al*, 2015), some degradation products of the Cas7-FLAG fusion protein are also detected .

Supplementary Table 1. Strains, plasmids and primers used.

A. Strains

**Strains**	**Characteristics**	**Reference/ source**
E. coli DH5α	F- ϕ80d*lacZ*ΔM15 Δ(*lacZYA-argF*) U169 *deoR recA*1 *endA*1 *hsdR*17(r_k^-, m_k^+) *gal-phoA supE*44λ- *thi*-1 *gyrA*96 *relA*1	Invitrogen (Thermo Fischer Scientific, Waltham, MA, USA)
E. coli GM121	F-, dam-3, dcm-6, ara-14, fhuA31, galK2, galT22, hdsR3, lacY1, leu-6, thi-1, thr-1, tsx-78	(Allers *et al*, 2010)
H119	DS70 (ΔpHV2), Δ*pyrE2*, Δ*trpA*, Δ*leuB*	(Allers *et al*, 2004)
Δ*cas8b* HV119	DS70 (ΔpHV2), Δ*pyrE2*, Δ*trpA*, Δ*leuB*,Δ*cas8b*	(Cass *et al*, 2015; Stoll, 2013)
*cas8*M545A HV120	DS70 (ΔpHV2), Δ*pyrE2*, Δ*trpA*, Δ*leuB, cas8b::cas8M545A*	this study

B. Plasmids

**Plasmids**	**Characteristics**	**Reference**
pTA131	ColE1 ori, f1 ori, lacZ, Amp^R, pyrE2,	(Allers *et al.*, 2004)
pTA131-up.Cas8M545A.do	ColE1 ori, f1 ori, lacZ, Amp^R, pyrE2, upstream (UP) and downstream (DO) region of HVO_A0206 (UP: 546 bp + *Cas8bM545A* + DO: 501 bp)	this study
pTA131-up.do(Csh1)	ColE1 ori, f1 ori, lacZ, Amp^R, pyrE2, upstream (UP) and downstream (DO) region of HVO_A0206 (UP: 546 bp + DO: 501 bp)	(Cass *et al.*, 2015)
pTA927-Cas7-N-FLAG	ColE1 ori, f1 ori, lacZ, Amp^R, pHV2 ori, p.*tna*-promoter, L11e+terminator, *pyrE2*, *cas7*N-FLAG	(Stoll, 2013)
pTA927-FLAGcontrol	ColE1 ori, f1 ori, lacZ, Amp^R, pHV2 ori, pyrE2, L11e-terminator, p.*tna* -promoter, 3xFLAG, t.Syn-terminator	(Wörtz *et al*, 2022)
pTA927	ColE1 ori, f1 ori, lacZ, Amp^R, pyrE2, pHV2 ori, p.*tnaA* promoter, t.syn terminator	(Allers *et al.*, 2010)
pTA927-FLAGC	ColE1 ori, f1 ori, lacZ, Amp^R,pyrE2, pHV2 ori, p.*tnaA* promoter, 3XFLAG, t.syn terminator	(Hadjeras *et al*, 2023)
pTA927-*cas11b*	ColE1 ori, f1 ori, lacZ, Amp^R, pHV2 ori, p.tna-Promotor, L11e+Terminator, pyrE2, Cas11b, t.syn terminator	this study

pTA927-*cas8b*-FLAGN	ColE1 ori, f1 ori, lacZ, Amp^R, pHV2 ori, p.*tna*-promoter, L11e+terminator, *pyrE2*, *cas8b*N-FLAG, t.syn terminator	(Cass *et al.*, 2015)
pTA927-*cas8b*-FLAGC	ColE1 ori, f1 ori, lacZ, Amp^R, pHV2 ori, p.*tna*-promoter, L11e+terminator, *pyrE2*, *cas8b*C-FLAG, t.syn terminator	this study
pTA927-*cas8b*M545A-FLAGC	ColE1 ori, f1 ori, lacZ, Amp^R, pHV2 ori, p.*tna*-promoter, L11e+terminator, *pyrE2*, *cas8bM545A*-C-FLAG, t.syn terminator	this study
pTA927-*cas8bM545A*-FLAGC-*cas11b*	ColE1 ori, f1 ori, lacZ, Amp^R, pHV2 ori, p.*tna*-promoter, L11e+terminator, *pyrE2*, *cas8b*C-FLAG, p.*tna*-promotor, *cas11b,* t.syn terminator	this study
pTA352	ColE1 ori, f1 ori, lacZ, Amp^R, leuB, pHV1/4 ori	(Norais *et al*, 2007)
pTA352-PAM3-P1.1	ColE1 ori, f1 ori, lacZ, Amp^R, leuB, pHV1/4 ori, PAM3 (TTC) followed by spacer 1 of CRISPR-locus P1)	(Maier *et al*, 2013)

C. Primers

**Oligonucleotide**	**Sequence**
Cas8_probe_do_rev	CGTCTTTATCGCTCGCCTCGAAGCTGAGC
Cas8_probe_intern_fwd	CGTGGGCCACCAGTTCACCGACTCG
Cas8_probe_do_fwd	TCCAACACATAACCAAACCAATGACGACACT
Cas8_probe_intern_rev	GGAACGATTCGAGTCTGTGTTCTCG
P1SP1	GTTCCGGGAGGTCGCCGGTCGAGATGCCTGC
5S	CGCAGGTGAGCTTAACTTCCGTGTTCGGG
5-Csh1-NdeI	TATTATCATATGACAGGTCCAGATATCGACGACTTC
3-Cas8-SmaI	TATTATCCCGGGGTTCGTGGTGCTCTCAGCGGGTTC
8M545A	TTCCAATCGAACCACCAACCGCGACAACCGAAACTGAAA
8M545Ar	TTTCAGTTTCGGTTGTCGCGGTTGGTGGTTCGATTGGAA
5-Cas11-NdeI	TATTATCATATGACAACCGAAACTGAAACTGAAACTGA
5-ptna-BamHI	TATTATGGATCCGCCCGTTCTCGTCGCGCTCTCGAAGCTGTT
3-Cas11-XbaI	TATTATTCTAGATTAGTTCGTGGTGCTCTCAGCGGGTTCTT
IP cas8 rev	CAGTCACTCGCCCGTGGAAGCG
IP cas8 fw	TCCAACACATAACCAAACCAATGACGACACT
3-cas8-rev	[phos]TTAGTTCGTGGTGCTCTCAGCGGGTT
5-cas8-fw	[phos]ATGACAGGTCCAGATATCGACGACTTC

Supplementary Table 2. Genes up- or down regulated on pHV4 in a Cas11 less strain. A few genes (e.g. HVO_A0344, A0345, A0348, A0365, A0370, A0389, A0390A, A0405) occur multiple times. These genes are disrupted (by transposon targeting, frameshift or in-frame stop codon), subregions are represented as distinct annotations, resulting them to be independently analysed. In this table only the top-scoring ones are shown, for the complete set please refer to the complete RNA-seq data (Supplementary Table 3).

A. Genes up regulated

gene	annotation	log2	pvalue	padj
HVO_A0508	1,2-phenylacetyl-CoA epoxidase subunit B, paaB	2.92	8.56036E-18	2.393E-16
HVO_A0510	DUF59 family protein	2.80	1.38984E-17	3.814E-16
HVO_A0509	1,2-phenylacetyl-CoA epoxidase subunit C, paaC	2.79	1.40757E-17	3.839E-16
HVO_A0507	1,2-phenylacetyl-CoA epoxidase subunit A, paaA	2.74	1.94817E-16	4.618E-15
HVO_A0535	peptidase M24 family protein	2.45	8.93135E-13	1.465E-11
HVO_A0505	enoyl-CoA hydratase, fadA4	2.04	1.03901E-15	2.329E-14

B. Genes down regulated

gene	annotation	log2	pvalue	padj
HVO_A0351	conserved hypothetical protein	-9.58	3.3884E-153	5.11E-150
HVO_A0348A	hypothetical protein	-9.21	1.7641E-144	2E-141
HVO_A0317	ArsR family transcription regulator	-9.19	4.5866E-172	2.08E-168
HVO_A0350	conserved hypothetical protein	-8.94	5.7363E-128	4.33E-125
HVO_A0386	N-methylhydantoinase (ATP-hydrolyzing) B, hyuB3	-8.44	9.8611E-138	8.93E-135
HVO_A0320	conserved hypothetical protein	-8.30	1.2137E-171	2.75E-168
HVO_A0331	D-galactonate dehydratase, dgoD1	-8.12	3.551E-118	2.01E-115
HVO_A0308	conserved hypothetical protein	-8.03	1.5489E-112	7.79E-110
HVO_A0334	conserved hypothetical protein	-7.91	2.8177E-109	1.28E-106
HVO_A0393	conserved hypothetical protein	-7.79	1.943E-99	6.768E-97
HVO_A0388	Lrp/AsnC family transcription regulator	-7.79	2.13784E-93	5.378E-91
HVO_A0318	conserved hypothetical protein	-7.75	6.99921E-97	2.113E-94
HVO_A0365	conserved hypothetical protein (nonfunctional)	-7.69	1.21826E-89	2.627E-87
HVO_A0315	conserved hypothetical protein	-7.59	6.71903E-91	1.521E-88
HVO_A0307	Lrp/AsnC family transcription regulator	-7.57	1.08273E-97	3.502E-95

HVO_A0380	ABC-type transport system periplasmic substrate-binding protein (probable substrate dipeptide/oligopeptide), dppA8	-7.48	1.9074E-121	1.23E-118
HVO_A0346	XerC/D-like integrase	-7.46	7.57704E-87	1.492E-84
HVO_A0279A	transcription elongation factor TFS, tfs3	-7.37	6.90862E-84	1.303E-81
HVO_A0385	N-methylhydantoinase (ATP-hydrolyzing) A, hyuA3	-7.32	4.5362E-104	1.71E-101
HVO_A0362	PQQ repeat protein	-7.31	5.00469E-96	1.416E-93
HVO_A0316	conserved hypothetical protein	-7.31	1.0843E-107	4.46E-105
HVO_A0369	CopG domain protein	-7.30	5.04342E-88	1.038E-85
HVO_A0397	HTH domain protein	-7.20	7.21604E-80	1.167E-77
HVO_A0374	conserved hypothetical protein	-7.17	5.55011E-78	8.377E-76
HVO_A0326	beta-D-galactosidase, bgaH	-7.14	8.2398E-94	2.195E-91
HVO_A0377	hydantoin racemase, hyuE	-7.00	1.3338E-82	2.237E-80
HVO_A0281	ABC-type transport system ATP-binding protein (probable substrate sugar), tsgD5	-6.97	3.36234E-78	5.25E-76
HVO_A0378	N-methylhydantoinase (ATP-hydrolyzing) B, hyuB2	-6.97	4.84794E-93	1.155E-90
HVO_A0329	2-dehydro-3-deoxy-phosphogluconate / 2-dehydro-3-deoxy-phosphogalactonate aldolase, bacterial-type, kdgA3	-6.90	8.38324E-72	1.116E-69
HVO_A0348	ISH7-type transposase ISHvo15 (nonfunctional)	-6.81	6.51126E-76	9.213E-74
HVO_A0401	Fido domain protein	-6.80	3.31882E-69	4.062E-67
HVO_A0368	RelE family protein	-6.70	8.07444E-67	8.705E-65
HVO_A0379	N-methylhydantoinase (ATP-hydrolyzing) A, hyuA2	-6.69	2.27991E-83	4.129E-81
HVO_A0394	HTH domain protein	-6.66	9.17271E-72	1.187E-69
HVO_A0288	probable oxidoreductase (short-chain dehydrogenase family)	-6.58	3.93232E-68	4.566E-66
HVO_A0291	conserved hypothetical protein	-6.54	1.12531E-68	1.341E-66
HVO_A0360	conserved hypothetical protein	-6.53	1.96585E-59	1.894E-57
HVO_A0376	probable Xaa-Pro dipeptidase, pepQ2	-6.47	1.30796E-73	1.795E-71
HVO_A0280	IclR family transcription regulator	-6.45	1.78399E-58	1.683E-56
HVO_A0333	SprT family protein	-6.44	1.48957E-62	1.499E-60
HVO_A0283	ABC-type transport system periplasmic substrate-binding protein (probable substrate sugar), tsgA5	-6.40	3.06666E-70	3.857E-68
HVO_A0347	conserved hypothetical protein (nonfunctional)	-6.31	2.23864E-55	2.027E-53
HVO_A0335	DUF1028 family protein	-6.30	9.79171E-60	9.638E-58
HVO_A0314	conserved hypothetical protein	-6.29	3.14575E-66	3.313E-64
HVO_A0332	IclR family transcription regulator GacR, gacR	-6.24	6.88362E-64	7.084E-62

HVO_A0311	probable halocin (homolog to halocin C8)	-6.23	1.12612E-67	1.244E-65
HVO_A0305	methylmalonate-semialdehyde dehydrogenase, mmsA	-5.94	8.07664E-53	6.772E-51
HVO_A0306	pyridoxal phosphate-dependent aminotransferase	-5.93	3.3734E-53	2.882E-51
HVO_A0287	homolog to mandelate racemase / homolog to muconate lactonizing enzyme	-5.88	3.64948E-54	3.178E-52
HVO_A0372	beta-lactamase domain protein	-5.85	4.45197E-49	3.417E-47
HVO_A0322	ABC-type transport system permease protein	-5.85	1.82096E-52	1.499E-50
HVO_A0312	HTH domain protein	-5.82	3.70162E-46	2.619E-44
HVO_A0339	ABC-type transport system periplasmic substrate-binding protein (substrate D-galactose), dppDF7	-5.82	5.96864E-68	6.756E-66
HVO_A0336	ABC-type transport system ATP-binding protein (substrate D-galactose), dppDF7	-5.81	3.99371E-58	3.691E-56
HVO_A0370	conserved hypothetical protein (nonfunctional)	-5.78	5.42146E-41	3.558E-39
HVO_A0313	cro/C1 family transcription regulator	-5.75	1.62746E-45	1.134E-43
HVO_A0396	SWIM zinc finger domain protein	-5.71	1.90646E-46	1.37E-44
HVO_A0286	DUF187 family protein	-5.71	1.06073E-46	7.747E-45
HVO_A0341	amidase (hydantoinase/carbamoylase family), amaB3	-5.68	6.38104E-50	4.982E-48
HVO_A0356	conserved hypothetical protein (nonfunctional)	-5.68	8.92677E-42	5.944E-40
HVO_A0330	D-galactose / L-arabinose dehydrogenase (NADP)	-5.67	5.99073E-42	4.049E-40
HVO_A0324	conserved hypothetical protein	-5.63	2.28056E-51	1.812E-49
HVO_A0387	cupin 2 barrel domain protein	-5.53	1.41147E-39	8.637E-38
HVO_A0289	enamine/imine deaminase, ridA4	-5.52	7.16183E-38	4.054E-36
HVO_A0398	conserved hypothetical protein	-5.47	9.6568E-39	5.622E-37
HVO_A0358	conserved hypothetical protein	-5.44	8.67E-48	6.436E-46
HVO_A0375	transcription initiation factor TFB, tfb11	-5.42	2.38667E-38	1.368E-36
HVO_A0345	transport protein (probable substrate cationic amino acids) (nonfunctional), cat4	-5.41	9.68465E-39	5.622E-37
HVO_A0392	death-on-curing family protein	-5.41	3.14936E-39	1.901E-37
HVO_A0384	ABC-type transport system ATP-binding protein (probable substrate dipeptide/oligopeptide), dppF8	-5.40	4.60384E-42	3.159E-40
HVO_A0402	conserved hypothetical protein	-5.39	1.0378E-39	6.619E-38
HVO_A0381	ABC-type transport system permease protein (probable substrate dipeptide/oligopeptide), dppB8	-5.35	5.74681E-49	4.337E-47

HVO_A0395	conserved hypothetical protein	-5.31	1.49597E-37	8.261E-36
HVO_A0323	ABC-type transport system ATP-binding protein	-5.31	7.28843E-39	4.342E-37
HVO_A0361	conserved hypothetical protein	-5.26	2.2435E-37	1.224E-35
HVO_A0328	2-keto-3-deoxygalactonate kinase, kdgK2	-5.24	2.28709E-34	1.204E-32
HVO_A0353	ISH3-type transposase ISH51 (nonfunctional)	-5.23	2.78738E-36	1.485E-34
HVO_A0344	UspA domain protein (nonfunctional)	-5.10	3.88392E-34	2.021E-32
HVO_A0338	ABC-type transport system permease protein (substrate D-galactose), dppB7	-5.03	3.57207E-40	2.311E-38
HVO_A0399	conserved hypothetical protein	-5.01	2.87257E-29	1.341E-27
HVO_A0383	ABC-type transport system ATP-binding protein (probable substrate dipeptide/oligopeptide), dppD8	-5.01	2.21141E-36	1.192E-34
HVO_A0296	probable oxidoreductase (short-chain dehydrogenase family)	-4.97	3.64332E-29	1.683E-27
HVO_A0389	HTH domain protein (nonfunctional)	-4.72	3.66704E-26	1.552E-24
HVO_A0371	conserved hypothetical protein	-4.71	6.99766E-33	3.482E-31
HVO_A0400	conserved hypothetical protein	-4.70	5.32566E-29	2.436E-27
HVO_A0403	ISHwa16-type transposase ISHvo16	-4.67	1.39402E-27	6.188E-26
HVO_A0342	IclR family transcription regulator	-4.64	2.13675E-30	1.018E-28
HVO_A0366	conserved hypothetical protein	-4.62	4.41338E-24	1.723E-22
HVO_A0310	conserved hypothetical protein	-4.54	1.86811E-24	7.42E-23
HVO_A0382	ABC-type transport system permease protein (probable substrate dipeptide/oligopeptide), dppC8	-4.51	6.32743E-27	2.729E-25
HVO_A0295	amidase (hydantoinase/carbamoylase family), amaB2	-4.40	3.56911E-24	1.405E-22
HVO_A0282	creatininase domain protein, cre3	-4.34	4.12131E-21	1.403E-19
HVO_A0357	conserved hypothetical protein	-4.22	2.91484E-20	9.427E-19
HVO_A0390A	conserved hypothetical protein (nonfunctional)	-4.20	5.84517E-20	1.851E-18
HVO_A0321	conserved hypothetical protein	-4.20	5.33652E-21	1.79E-19
HVO_A0299	ABC-type transport system periplasmic substrate-binding protein	-4.12	5.49814E-20	1.753E-18
HVO_A0290	2-dehydro-3-deoxy-phosphogluconate aldolase, bacterial-type, kdgA2	-4.10	8.7687E-20	2.738E-18
HVO_A0303	probable allantoinase, pucH2	-4.08	1.48151E-22	5.59E-21
HVO_A0297	ABC-type transport system permease protein	-4.05	1.18198E-17	3.263E-16
HVO_A0290A	2-keto-3-deoxygluconate kinase (nonfunctional), kdgK3	-4.01	2.26496E-18	6.45E-17
HVO_A0284	ABC-type transport system permease protein (probable substrate sugar), tsgB5	-3.96	5.40935E-19	1.633E-17

HVO_A0337	ABC-type transport system permease protein (substrate D-galactose), dppC7	-3.93	1.39744E-22	5.317E-21
HVO_A0389	HTH domain protein (nonfunctional)	-3.87	6.30346E-17	1.586E-15
HVO_A0319	hypothetical protein	-3.79	2.37303E-15	5.045E-14
HVO_A0327	conserved hypothetical protein	-3.78	2.27274E-15	4.854E-14
HVO_A0294	ABC-type transport system ATP-binding protein	-3.39	6.52512E-14	1.211E-12
HVO_A0301	probable polysaccharide deacetylase	-3.32	3.17828E-12	4.797E-11
HVO_A0390A	conserved hypothetical protein (nonfunctional)	-3.31	5.06546E-12	7.399E-11
HVO_A0391	conserved hypothetical protein	-3.26	3.8727E-11	4.954E-10
HVO_A0405	conserved hypothetical protein (nonfunctional)	-3.23	9.56218E-11	1.167E-09
HVO_A0405	conserved hypothetical protein (nonfunctional)	-3.10	4.88594E-11	6.18E-10
HVO_A0292	transport protein (probable substrate cationic amino acids), cat5	-3.09	5.14714E-11	6.474E-10
HVO_A0293	ABC-type transport system ATP-binding protein	-3.08	2.62298E-12	4.012E-11
HVO_A0298	probable oxidoreductase (short-chain dehydrogenase family)	-2.66	6.86847E-08	5.236E-07
HVO_A0325	UPF0121 family protein	-2.61	2.20532E-07	1.541E-06
HVO_A0285	ABC-type transport system permease protein (probable substrate sugar), tsgC5	-2.53	6.64276E-07	4.224E-06
HVO_A0389	HTH domain protein (nonfunctional)	-2.45	1.391E-06	8.366E-06
HVO_A0302	asparaginase/glutaminase family protein, ansB	-2.44	2.9145E-07	2E-06
HVO_A0023	DUF1931 domain protein	-2.43	4.18319E-20	1.343E-18
HVO_A0363	conserved hypothetical protein	-2.42	7.1988E-07	4.559E-06
HVO_A0300	ABC-type transport system permease protein	-2.41	1.60136E-07	1.149E-06
HVO_A0295A	luciferase family protein	-2.38	2.26198E-06	1.305E-05
HVO_A0359	conserved hypothetical protein (nonfunctional)	-2.22	1.32925E-05	6.451E-05
HVO_A0309	ISH7-type transposase HfIRS6 (nonfunctional)	-2.16	2.20387E-05	0.0001016

References

Abramson J, Adler J, Dunger J, Evans R, Green T, Pritzel A, Ronneberger O, Willmore L, Ballard AJ, Bambrick J *et al* (2024) Accurate structure prediction of biomolecular interactions with AlphaFold 3. *Nature* 630: 493-500

Allers T, Barak S, Liddell S, Wardell K, Mevarech M (2010) Improved strains and plasmid vectors for conditional overexpression of His-tagged proteins in Haloferax volcanii. *Appl Environ Microbiol* 76: 1759-1769

Allers T, Ngo HP, Mevarech M, Lloyd RG (2004) Development of additional selectable markers for the halophilic archaeon Haloferax volcanii based on the leuB and trpA genes. *Appl Environ Microbiol* 70: 943-953

Brendel J, 2014. Charakterisierung der Prozessierungs- und Interferenzaktivität des CRISPR/Cas-Systems in Haloferax volcanii, Faculty of Natural Sciences. Ulm University, Ulm.

Cass SD, Haas KA, Stoll B, Alkhnbashi O, Sharma K, Urlaub H, Backofen R, Marchfelder A, Bolt EL (2015) The role of Cas8 in type I CRISPR interference. *Biosci Rep* 5: e00197

Guan Y, Zhu Q, Huang D, Zhao S, Jan Lo L, Peng J (2015) An equation to estimate the difference between theoretically predicted and SDS PAGE-displayed molecular weights for an acidic peptide. *Scientific Reports* 5: 13370

Hadjeras L, Bartel J, Maier LK, Maaß S, Vogel V, Svensson SL, Eggenhofer F, Gelhausen R, Müller T, Alkhnbashi OS *et al* (2023) Revealing the small proteome of Haloferax volcanii by combining ribosome profiling and small-protein optimized mass spectrometry. *Microlife* 4: uqad001

Maier LK, Lange SJ, Stoll B, Haas KA, Fischer S, Fischer E, Duchardt-Ferner E, Wohnert J, Backofen R, Marchfelder A (2013) Essential requirements for the detection and degradation of invaders by the Haloferax volcanii CRISPR/Cas system I-B. *RNA biology* 10: 865-874

Norais C, Hawkins M, Hartman AL, Eisen JA, Myllykallio H, Allers T (2007) Genetic and physical mapping of DNA replication origins in Haloferax volcanii. *PLoS Genet* 3: e77. Epub 2007 Apr 2005.

Stoll B, 2013. Analyse des prokaryotischen Immunsystems CRISPR/Cas Typ I-B im archaealen Modellorganismus Haloferax volcanii, Biology II. Ulm University, Ulm.

Wörtz J, Smith V, Fallmann J, König S, Thuraisingam T, Walther P, Urlaub H, Stadler PF, Allers T, Hille F *et al* (2022) Cas1 and Fen1 Display Equivalent Functions During Archaeal DNA Repair. *Frontiers in Microbiology* 13
